# Creation of Simulation-Based Curriculum of Perioperative Emergencies for Residents in Anesthesiology

**DOI:** 10.7759/cureus.15509

**Published:** 2021-06-07

**Authors:** Michael R Kazior, Stefan Ianchulev, Jonathan Nguyen, Brooke Trainer-Albright, Paras Shah

**Affiliations:** 1 Anesthesiology, Central Virginia Veterans Affairs Medical Center, Richmond, USA; 2 Anesthesiology and Critical Care, Virginia Commonwealth University School of Medicine, Richmond, USA; 3 Medical Education and Simulation, Central Virginia Veterans Affairs Medical Center, Richmond, USA

**Keywords:** simulation in medical education, crisis management, academic anesthesiology, simulation education, operating room

## Abstract

Introduction: Crisis management is difficult to practice and evaluate for resident learners and leadership given the rarity of these events in clinical practice. However, simulation provides a medium to bridge this gap. We identified a need for simulation in our anesthesiology residency program to help residents learn to treat perioperative emergencies.

Objective: To describe the process of curriculum development, content, and early outcomes of a simulation-based curriculum for the management of perioperative emergencies for all levels of anesthesiology learners.

Materials and methods: Curriculum development began in the Spring of 2019 and simulations began in August 2019. All anesthesiology residents rotating at a single center through December 2020 were eligible. Each resident was given their own simulation scenario detailing a specific perioperative emergency and then debriefed as a group afterward. All residents participating in the scenario were given a post-simulation survey assessing the value of the educational experience, relevance to their level of training, and quality of learning environment.

Results: Out of 90 eligible residents, 79 participated in the study (87%). Overall, 100% of participants completed the post-simulation survey; 100% of residents reported that the curriculum was useful to their education; 98% of residents reported that the curriculum was relevant to their training level; 99% of residents reported that the simulation was an engaging learning experience.

Conclusion: A simulation-based curriculum of perioperative emergencies for anesthesiology residents is feasible to implement, viewed as worthwhile by trainees, and can foster education in a different learning environment.

## Introduction

The use of simulation-based training in graduate medical training programs has become widespread over the last decade [1-2]. This is especially true in the field of anesthesiology, where simulation-based education is used as a method to prepare trainees to handle perioperative emergencies that happen so rarely they may not experience them in clinical practice during their training [3-5]. An anesthesiology residency program that offers simulation, in combination with a traditional curriculum involving didactic lectures and clinical time, can better prepare trainees to be independent and comfortable managing any perioperative pathology [5].

The Accreditation Council for Graduate Medical Education (ACGME) has affirmed the value of simulation in anesthesiology education by including it in the Milestones project, which is used to evaluate residents’ progress through residency [6-8]. The American Board of Anesthesiology (ABA) has echoed this sentiment by adding the Objective Structured Clinical Examination (OSCE) to the requirement for board certification [7-9]. High fidelity simulation helps faculty evaluate residents along the milestones, provides residents a safe environment to learn, and introduces them to the modality they will be tested on during the OSCE [7-9].

In the fall of 2018, the need for high fidelity simulation in the educational experience for anesthesiology residents was identified at our institution. To address this educational need, we proposed a simulation-based curriculum for the management of perioperative emergencies for all levels of training. We aim to describe the process of curriculum development, content, and early outcomes of the first eighteen months of implementation. This data was previously presented as a meeting abstract at the 2021 International Anesthesia Research Society Meeting on May 15, 2021.

## Materials and methods

The Central Virginia Veterans Affairs Health Care System (VA) is an affiliate site for the training of residents in anesthesiology from Virginia Commonwealth University (VCU) Health. The Department of Anesthesiology at VCU Health has fourteen residents in each of the categorical anesthesia years, post-graduate year (PGY)-2 through PGY-4. The VA hosts five residents every rotation block from this pool; three PGY-2 residents, one PGY-3 resident, and one PGY-4 resident.

A group of stakeholders from the Department of Anesthesiology came together to develop the curriculum in the spring of 2019. The goals for this experience were to create a novel educational experience for the residents, be relevant to the residents at their specific level of training, and to provide a non-judgmental environment to facilitate learning.

To ensure a novel experience, we developed the curriculum around perioperative emergencies. Other than traditional didactics and clinical experience, nothing prepared the residents for these in the residency program. Simulation is the perfect environment for learning without the threat of real patient harm. This also meets several ACGME anesthesiology milestones, primarily Situational Awareness and Crisis Management (Patient Care [PC] 7), but also the Application and Interpretation of Monitors (PC3), Intra-Operative Care (PC4), Airway Management (PC5), and Post-Operative Care (PC8). 

Scenarios for the PGY-2 residents included anaphylaxis, malignant hyperthermia, and myocardial infarction. Scenarios for the upper level residents (PGY-3 and PGY-4) included venous air embolism, local anesthetic toxicity, and obstetric hemorrhage.

Each resident was given their own scenario and entered the simulated environment while the other residents watched from the debriefing room and could be called in to help if needed. Our simulation center employs the SimMan 3G (Laerdal Medical, Wappingers Falls, NY) and utilizes the ASL 5000 Breathing Simulator (IngMar Medical, Pittsburgh, PA) to give better functionality with the anesthesia machine. A Standard Operating Guide (SOG) was utilized for each scenario for uniformity.

Each simulation scenario ended with a formal debriefing with the simulation faculty and all participating residents. Debriefing was at the discretion of each faculty member but the Debriefing with Good Judgement model was encouraged [10]. This allowed faculty to assess the ACGME resident milestones of Reflective Practice and Commitment to Personal Growth (Practice Based Learning and Improvement [PBLI] 2), Interprofessional and Team Communication (Interpersonal and Communication Skills [ICS] 2), Foundational Knowledge (Medical Knowledge [MK] 1), and Clinical Reasoning (MK2).

**Figure 1:**
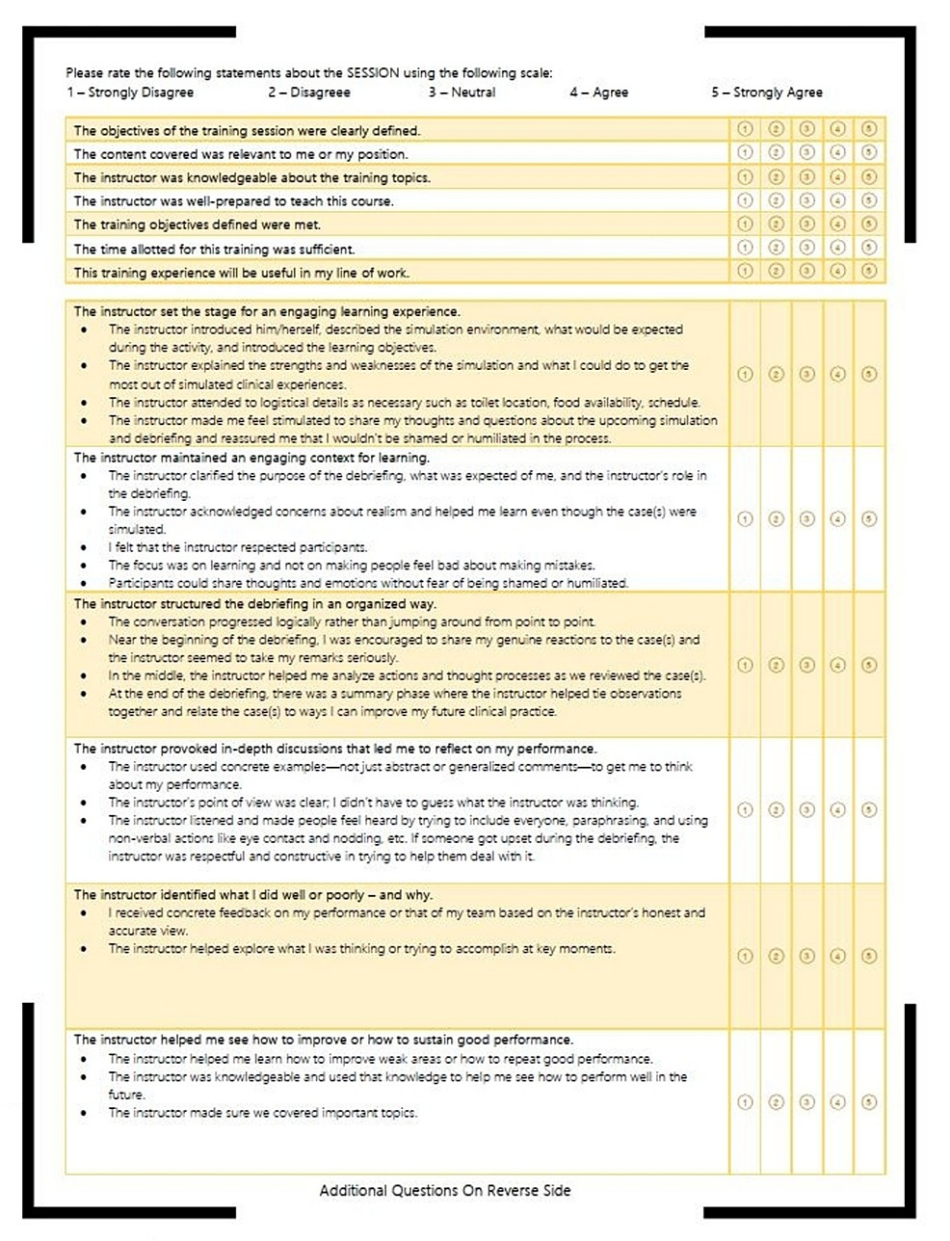
Post-Simulation Survey Page 1

**Figure 2:**
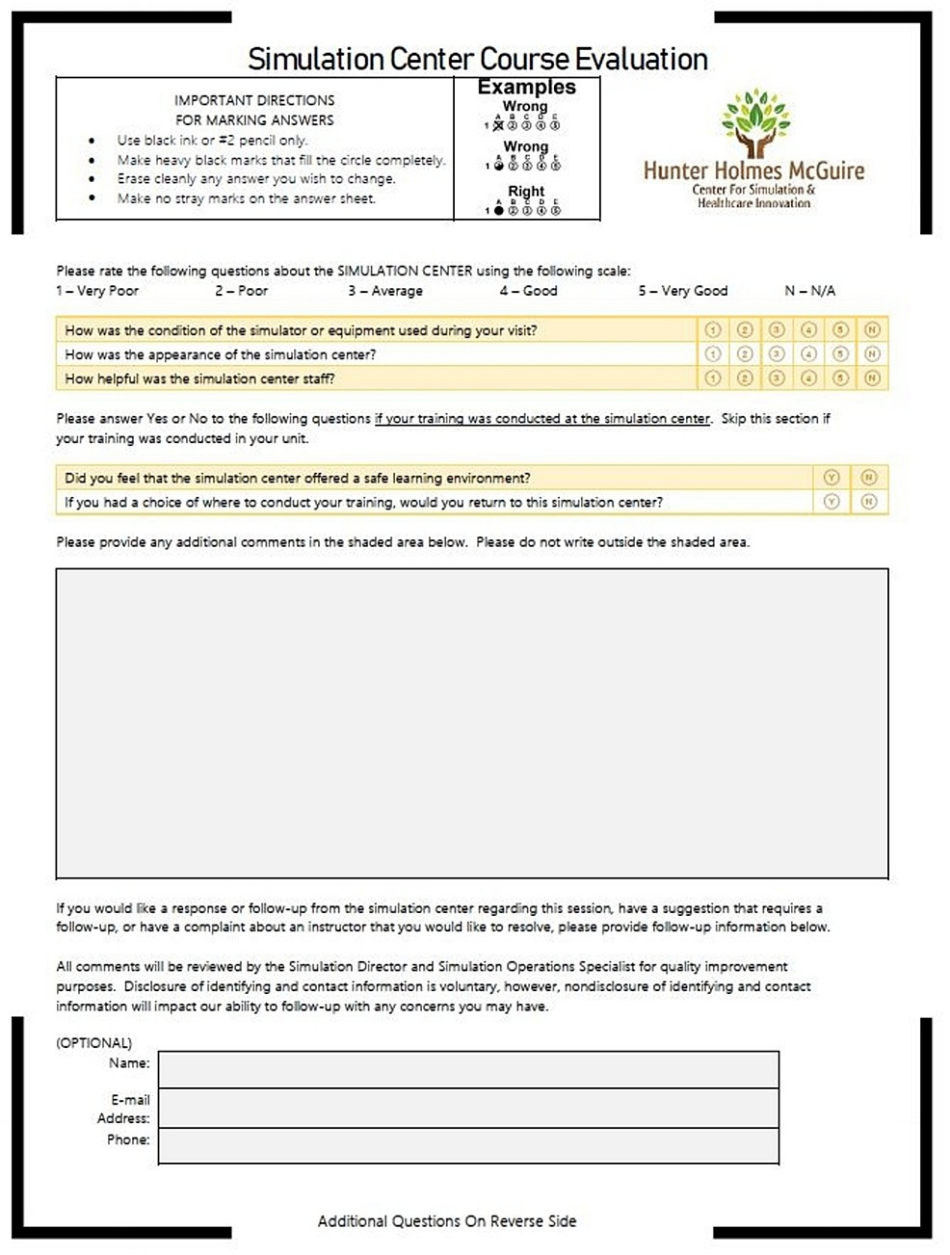
Post-Simulation Survey Page 2

At the end of the simulation session, each resident filled out a survey (supplemental) to give feedback on the new curriculum on a 5-point Likert scale (1= strongly disagree, 2= disagree, 3= neutral, 4= agree, 5= strongly agree). Additional comments were welcomed. Study questions sought to assess the relevance of the curriculum and the quality of the learning environment (Figures 1-2). This survey was developed in house at the VA for this purpose with no collection of validity evidence. This study was exempt from review by the VA Institution Review Board (IRB). 

## Results

The first simulations under this new curriculum began in August 2019 and are still currently running as of March 2021. Data presented is through December 2020. Out of 90 eligible residents rotating at the VA during this time period, 79 participated in the study (87%). There was a three-month break from March 2020 to June 2020 out of an abundance of caution due to the COVID-19 pandemic. During this time period, we completed nineteen simulation sessions involving seventy-nine residents for a total of three-hundred fifty-nine resident training hours. 

**Figure 3:**
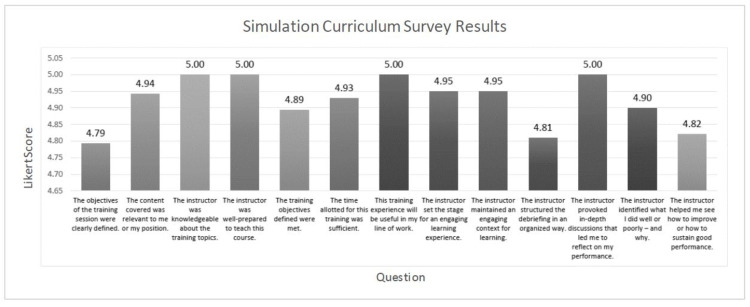
Simulation Curriculum Results The above questions were present on a post-simulation survey the residents were asked to fill out. A 5-point Likert scale (1= strongly disagree, 2= disagree, 3= neutral, 4= agree, 5= strongly agree) was used to score the feedback. Average response values are seen above each bar.

Several survey responses recorded an average Likert scale of 5.00 and included “the instructor was knowledgeable about the training topics”, “the instructor was well-prepared to teach this course”, “this training experience will be useful in my line of work”, and “the instructor provoked in-depth discussions that led me to reflect on my performance.” The response that received the lowest Likert score was “the objectives of the training session were clearly defined” with a 4.79 score (Figure 3).

Additional positive comments provided by participants included “it was a good learning environment, low pressure and educational” and “I felt comfortable throughout, I did not feel like I was being judged, it was a safe space to discuss the cases, the scenarios will help me be more comfortable in the operating room.” Additional negative feedback received included “frantic at times, slightly confused about the different ventilator that I am not used to.” (Table 1).

**Table 1:** Additional Comments Provided on Simulation Curriculum

Comment:
It was a good learning environment, low pressure and educational.
The simulation experiences, both low and high fidelity, were realistic, timely, and relevant to anesthesia bootcamp. Instructors were encouraging and approachable, and stress was minimal, which all promoted learning.
It was very fun and good learning. High fidelity. We should do more.
Frantic at times. Slightly confused about a different ventilator than I am used to. Overall comfortable with the simulation.
I felt comfortable throughout. I did not feel like I being judged. It was a safe space to discuss the cases. These scenarios will help me be more comfortable in the OR.
I was able to learn and further tune my knowledge base and skills which at the end of the day had a positive impact on my confidence.
very educational experience good team building, motivated and challenged.
It was a great experience. Very fun and useful for providing how to work through problems in the OR as well as the educational debriefings.
I thought the simulation was appropriate for our current level of training and allowed us to trouble shoot the problems in a safe and educational environment.
Encouraged to keep learning.
Great facility, excellent learning opportunities.
Beneficial debriefing and not only going over the medicine aspects of the sim, but what steps should be taken to stabilize the patient / grabbing help sooner.

## Discussion

The high level of resident participation shows the creation of a novel educational program. The post-simulation survey results provide evidence that we created a curriculum that was relevant to the residents and an educational environment where they could effectively learn.

We learned a lot throughout this process of building a simulation program at an affiliate educational site. One of the biggest hurdles was getting the residents out of clinical duties without disrupting workflow in the operating rooms. Having support from departmental leadership to provide non-clinical time for faculty and residents is essential. It also takes buy in from faculty colleagues to participate in the simulation and support the changes to workflow.

The technical difficulties and troubleshooting underlined the importance of having an experienced and reliable simulation operations specialist. Without this support the simulation curriculum product would not have been as streamlined or simply would not have happened.

The main limitation of this educational project is that it is a small, single-center review which limits generalizability. No pre-curriculum test was performed in this study which also limits further statistical analysis of the data. Evaluation by the residents who participated in these simulations only assessed level one Kirkpatrick [11] scores and the evaluation survey was not tested for validity. Given the nascent stages of the project, we felt that this represented the best place to start because we were primarily concerned with feasibility.

We plan on continuing this simulation program and want to grow by developing new scenarios for the perioperative emergency curriculum. As the curriculum is developed, we seek to incorporate more rigorous data collection, assess higher Kirkpatrick level scores, and incorporate pre and post-simulation surveys. Developing better feedback and evaluations to assess the higher Kirkpatrick scores will be useful for elucidating the effect that this education has on resident performance and patient outcomes. Simulation can also help supplement many more areas of resident education and assess their progression through training based on the ACGME milestones.

## Conclusions

Preliminary data shows the simulation-based curriculum around perioperative emergencies met our goals. With the break in simulation activities for COVID-19, more than one simulation session occurred every month and all the anesthesia residents in the residency program had exposure to simulation. This shows that we met our goal of creating a novel educational program that did not exist prior to this venture. The post-simulation survey results provide evidence that we achieved our other goals of creating a curriculum that was relevant to these learners and that they had an educational environment where they could effectively learn the material. Future directions are to develop new scenarios for perioperative emergency curriculum, create new curricula for different resident education needs (advanced cardiovascular life support in the operating room), and determine a better way to evaluate residents after they have participated in the simulation.
